# Supplementary material for: Impaired semen quality, an increase of sperm morphological defects and DNA fragmentation associated with environmental pollution in urban population of young men from Western Siberia, Russia
Source: PLoS One. 2021 Oct 22;16(10):e0258900. doi: 10.1371/journal.pone.0258900 (PMC8535459; doi:10.1371/journal.pone.0258900)
Supplement: S1 Table — Bold text indicates significant (p<0.05) influences showed by ANCOVA. SQS–sperm quality status: normozoospermia (sperm concentration and percentage of progressively motile spermatozoa equal to or above the lower reference limits) and pathozoospermia (sperm concentration < 15 mln/ml and/or percentage of progressively motile spermatozoa<32%). ERC–excess residual cytoplasm. (DOCX) [file pone.0258900.s001.docx]

**S1 Table**

The influence of sperm quality status and region on DFI and percentages of sperm morphology defects (ANCOVA results).

|  |  | Factors | |  | | |  |
| --- | --- | --- | --- | --- | --- | --- | --- |
|  | City |  | SQS |  | | SQS&City |  |
| Parameters |  |  |  |  | |  |  |
|  | F criterion | p value | F criterion | p value | | F criterion | p value |
| DFI | 1.63096 | 0.202737 | 25.24645 | **0.000001** | | 5.48586 | **0.019944** |
| Normal sperm % | 16.7234 | **0.000051** | 238.7347 | **<0.00001** | | 0.0246 | 0.875374 |
| TZI | 43.423 | **<0.00001** | 143.721 | **<0.00001** | | 0.396 | 0.529387 |
| Head abnormalities | | | | | | | |
| Amorphous, % | 112.7628 | **0.000001** | 0.8672 | 0.352203 | | 7.5839 | **0.006111** |
| Pyriform, % | 37.15174 | **<0.00001** | 3.38163 | 0.066539 | | 4.40574 | **0.036336** |
| Elongated, % | 44.10903 | **<0.00001** | 6.32328 | **0.012239** | | 1.00441 | 0.316746 |
| Round, % | 42.41449 | **<0.00001** | 4.47411 | **0.034922** | | 1.05763 | 0.304270 |
| Large, % | 1.052657 | 0.305408 | 1.229752 | 0.268006 | | 0.134328 | 0.714147 |
| Small, % | 6.76772 | **0.009566** | 5.72077 | **0.017146** | | 0.98016 | 0.322655 |
| Double, % | 3.366600 | 0.067144 | 7.795593 | **0.005445** | | 0.241969 | 0.623010 |
| Vacuolated, % | 27.5218 | **<0.00001** | 7.3285 | **0.007027** | | 8.9148 | **0.002972** |
| Abnormal acrosome, % | 6.8434 | **0.009175** | 82.4790 | **<0.00001** | | 0.0001 | 0.993432 |
| Midpiece abnormalities | | | | | | | |
| Bent_head, % | 0.0043 | 0.947767 | 46.4374 | **<0.00001** | | 0.8376 | 0.360535 |
| Asymmetrical neck insertion, % | 359.5111 | **<0.00001** | 2.0104 | 0.156869 | | 8.5968 | **0.003527** |
| Thick mipiece, % | 4.7220 | **0.030263** | 17.0368 | **0.000043** | | 1.9884 | 0.159148 |
| Thin midpiece, % | 2.79078 | 0.095455 | 19.05750 | **0.000016** | | 1.94909 | 0.163325 |
| Tail abnormalities | | | | | | | |
| Double tail, % | 0.01202 | 0.912735 | 6.22455 | **0.012931** | | 0.15577 | 0.693258 |
| Coiled tail,% | 0.2300 | 0.631714 | 43.9793 | **<0.00001** | | 2.3579 | 0.125301 |
| Short tail, % | 5.0583 | **0.024957** | 107.5698 | **<0.00001** | | 0.6007 | 0.438684 |
| Excess residual cetoplasm | | | | | | | |
| ERC, % | 11.9886 | **0.000583** | 22.1720 | | **0.000003** | 5.3240 | **0.021455** |
| Abnormalities in different parts of spermatozoon | | | | | | | |
| Head, % | 141.445 | **<0.00001** | 52.868 | | **<0.00001** | 0.001 | 0.974849 |
| Midpiece,% | 59.37937 | **<0.00001** | 51.37408 | | **<0.00001** | 1.29904 | 0.254952 |
| Tail, % | 13.42102 | **0.000276** | 10.07110 | | **<0.00001** | 0.00482 | 0.944661 |
| Head&Midpiece_% | 167.7070 | **<0.00001** | 57.8435 | | **<0.00001** | 1.7336 | 0.188573 |
| Head&Tail_% | 0.0662 | 0.797011 | 65.5943 | | **<0.00001** | 1.2420 | 0.265638 |
| Midpiece&Tail_% | 8.825140 | **0.003119** | 0.032874 | | 0.856198 | 0.129713 | 0.718887 |
| Head&Midpiece&Tail_% | 1.1478 | 0.284545 | 127.6329 | | **<0.00001** | 0.0075 | 0.930926 |

*Note.*

Significant (p<0.05) effects of factors are highlighted by bold text.

Abbreviations: DFI – DNA fragmentation index; TZI – teratozoospermia index; ERC – excess residual cytoplasm. SQS – sperm quality status (normal semen parameters or pathozoospermia).
