# Supplementary material for: Impaired semen quality, an increase of sperm morphological defects and DNA fragmentation associated with environmental pollution in urban population of young men from Western Siberia, Russia
Source: PLoS One. 2021 Oct 22;16(10):e0258900. doi: 10.1371/journal.pone.0258900 (PMC8535459; doi:10.1371/journal.pone.0258900)
Supplement: S2 Table — Results based on raw data. Analysis of variance used to compare all parameters. Significant (p<0.05) differences between groups are highlighted by bold text. Abbreviations: SD—standard deviation; (5–95) - 5th–95th percentile; DFI–DNA fragmentation index; TZI–teratozoospermia index; ERC–excess residual cytoplasm. (DOCX) [file pone.0258900.s002.docx]

**S2 Table** The Relationship between Semen Quality And Sperm Morphology

|  |  | Sperm quality |  |  |  |
| --- | --- | --- | --- | --- | --- |
| Parameters | Normal(n=347) |  | Decreased (n=188) | | P value |
|  | Mean(SD) | Median(5-95) | Mean(SD) | Median(5-95) |  |
| Normal sperm % | **8.55(2.7)** | 8.50(4.50-13.75) | **4.75(2.35)** | 4.50(1.25-8.75) | **0.000009** |
| TZI | **1.44(0.09)** | 1.44(1.30-1.60) | **1.57(0.14)** | 1.55(1.38-1.82) | **0.000009** |
| DFI | **7.65(5.62)** | 6.17(2.40-19.71) | **14.07(9.54)** | 11.42(3.72-35.64) | **0.000009** |
| Amorphous head,.% | 63.38(13.16) | 64.50(39.00-82.50) | 62.35(17.23) | 65.50(30.50-84.5) | 0.653099 |
| Pyriform head, % | **8.65(8.74)** | 6.50(0.50-27.50) | **11.68(12.18)** | 7.00(0.5-39.42) | **0.008153** |
| Elongated head, % | **10.2(8.55)** | 8.00(1.00-27.50) | **13.2(10.40)** | 10.51(1.5-32.5) | **0.000544** |
| Round head, % | **1.48(2.07)** | 1.00(0.00-5.00) | **2.01(2.24)** | 1.00(0.00-7.50) | **0.001968** |
| Large head, % | 0.12(0.26) | 0.00(0.00-0.50) | 0.16(0.36) | 0.00(0.00-0.50) | 0.347563 |
| Small head, % | **0.5(0.81)** | 0.00(0.00-2.00) | **0.66(0.87)** | 0.50(0.00-2.50) | **0.040066** |
| Double head, % | **0.03(0.13)** | 0.00(0.00-0.50) | **0.08(0.29)** | 0.00(0.00-0.50) | **0.009677** |
| Vacuolated head, % | **10.06(6.36)** | 9.00(2.00-22.00) | **12.35(7.83)** | 11.00(2.50-29.00) | **0.001192** |
| Acrosome defects, % | **14.64(8.25)** | 12.50(5.50-32.50) | **23.86(13.41)** | 21.25(7.50-52.00) | **0.000009** |
| Bent head_% | **4.89(3.30)** | 4.00(1.00-10.50) | **7.63(4.85)** | 6.50(2.00-16.83) | **0.000009** |
| ERC_% | **6.98(3.99)** | 6.00(1.50-14.25) | **9.19(5.42)** | 8.00(2.50-18.00) | **0.000009** |
| Asymmetrical neck insertion_% | **17.62(8.31)** | 16.75(6.00-32.50) | **19.55(7.66)** | 19.50(7.50-32.50) | **0.000016** |
| Thick_mipiece_% | **6.23(3.53)** | 6.00(2.00-13.00) | **7.82(3.87)** | 7.50(2.50-14.50) | **0.000012** |
| Thin_midpiece_% | **0.81(0.82)** | 0.50(0.00-2.50) | **1.45(1.66)** | 1.00(0.00-4.50) | **0.000011** |
| Double_tail_% | **1.19(1.12)** | 1.00(0.00-3.00) | **1.51(1.32)** | 1.00(0.00-4.00) | **0.014916** |
| Coiled_tail_% | **9.10(4.98)** | 8.00(3.50-19.50) | **12.55(6.76)** | 11.50(4.50-26.50) | **0.000009** |
| Short-tail_% | **1.87(1.46)** | 1.50(0.00-4.50) | **3.79(2.83)** | 3.00(0.50-10.50) | **0.000009** |
| Abnormalities in different parts of spermatozoon | | | | | |
| Head_% | **48.53(8.91)** | 48.00(35.00-63.00) | **41.65(10.36)** | 41.50(25.33-58.0) | **0.000009** |
| Midpiece_% | **4.53(2.95)** | 4.00(1.00-10.00) | **3.21(2.54)** | 2.50(0.00-8.50) | **0.000009** |
| Tail_% | **1.47(1.46)** | 1.00(0.00-4.00) | **1.08(1.27)** | 1.00(0.00-3.50) | **0.000145** |
| Head&Midpiece_% | **26.40(7.72)** | 25.00(15.50-39.50) | **32.64(8.63)** | 32.00(18.00-47.5) | **0.000009** |
| Head&Tail_% | **7.93(3.88)** | 7.50(3.00-15.50) | **11.46(6.14)** | 11.00(4.00-23.00) | **0.000009** |
| Midpiece&Tail_% | 0.27(0.44) | 0.00(0.00-1.00) | 0.32(0.50) | 0.00(0.00-1.50) | 0.566345 |
| Head&Midpiece&Tail_% | **2.45(1.62)** | 2.00(0.50-5.50) | **4.95(3.07)** | 4.00(1.50-10.50) | **0.000009** |

*Note.* Results based on raw data. Analysis of variance used to compare all parameters. Significant (p<0.05) differences between groups are highlighted by bold text.

Abbreviations: SD - standard deviation; (5–95) - 5th–95th percentile; DFI – DNA fragmentation index; TZI – teratozoospermia index; ERC – excess residual cytoplasm.
