# Supplementary material for: Impaired semen quality, an increase of sperm morphological defects and DNA fragmentation associated with environmental pollution in urban population of young men from Western Siberia, Russia
Source: PLoS One. 2021 Oct 22;16(10):e0258900. doi: 10.1371/journal.pone.0258900 (PMC8535459; doi:10.1371/journal.pone.0258900)
Supplement: S3 Table — Significant (p<0.05) effects of factors are highlighted by bold text. Abbreviations: DFI–DNA fragmentation index; TZI–teratozoospermia index; ERC–excess residual cytoplasm. (DOCX) [file pone.0258900.s003.docx]

**S3 Table.**

**The influence of DNA fragmentation level on sperm quality and percentage of sperm morphology defects (ANCOVA results).**

|  |  |  | | Factors | |  |
| --- | --- | --- | --- | --- | --- | --- |
|  | City |  | DFI level |  | DFI level&City interactions |  |
| Parameters |  |  |  |  |  |  |
|  | F criterion | p value | F criterion | p value | F criterion | p value |
| Sperm count, mln | 1.68215 | 0.195830 | 13.06174 | **0.000364** | 9.09571 | **0.002825** |
| Sperm concentration, mln/ml | 4.42943 | 0.036319 | 24.06476 | **0.000002** | 10.50275 | **0.001353** |
| Progressive motility, % | 0.11494 | 0.734873 | 42.45741 | **<0.00001** | 2.45426 | 0.118466 |
| Normal sperm, % | 1.38332 | 0.240650 | 20.45226 | **0.000009** | 0.99001 | 0.320699 |
| TZI | 23.339 | **<0.00001** | 11.074 | **0.001007** | 3.821 | 0.051711 |
| DFI, % | 1.8982 | 0.169510 | 456.3640 | **<0.00001** | 0.1976 | 0.657016 |
| Amorphous head, % | 27.3857 | **<0.00001** | 2.1464 | 0.144160 | 0.3098 | 0.578320 |
| Pyriform head, % | 3.48917 | 0.062937 | 0.12435 | 0.724663 | 0.16968 | 0.680747 |
| Elongated head, % | 44.10903 | **<0.00001** | 6.32328 | **0.012239** | 1.00441 | 0.316746 |
| Round head, % | 21.17249 | **0.000007** | 0.02061 | 0.885959 | 0.21162 | 0.645896 |
| Large head,, % | 0.551285 | 0.458487 | 0.667685 | 0.414635 | 0.601557 | 0.438715 |
| Small head, % | 0.676062 | 0.411726 | 0.320013 | 0.572105 | 0.374018 | 0.541376 |
| Double head,, % | 10.97767 | **0.001058** | 8.57765 | **0.003716** | 5.87729 | **0.016044** |
| Vacuolated head,, % | 9.36512 | **0.002452** | 0.50548 | 0.477764 | 0.00874 | 0.925591 |
| Abnormal acrosome, % | 0.38237 | 0.536900 | 15.29489 | **0.000118** | 1.79201 | 0.181893 |
| Bent_head, % | 4.06170 | **0.044932** | 17.23153 | **0.000045** | 1.21582 | 0.271238 |
| ERC, % | 1.60005 | 0.207069 | 0.00000 | 1.000000 | 1.87486 | 0.172144 |
| Asymmetrical neck insertion, % | 156.7867 | **<0.00001** | 0.2021 | 0.653410 | 0.9890 | 0.320933 |
| Thick mipiece, % | 0.35789 | 0.550222 | 0.05474 | 0.815198 | 2.98486 | 0.085277 |
| Thin midpiece, % | 3.874582 | 0.050123 | 0.949918 | 0.330679 | 1.184885 | 0.277408 |
| Double tail, % | 0.18180 | 0.670192 | 0.07938 | 0.778377 | 1.12711 | 0.289413 |
| Coiled tail,% | 1.62655 | 0.203360 | 13.57387 | **0.000281** | 3.05828 | 0.081549 |
| Short tail, % | 3.05828 | 0.081549 | 5.66307 | **0.018073** | 0.62505 | 0.429923 |
| **Abnormalities in different parts of spermatozoon** | | | | | | |
| Head, % | 52.5102 | **<0.00001** | 6.3782 | **0.012170** | 7.5674 | **0.006376** |
| Midpiece,% | 21.60569 | **0.000005** | 8.61710 | **0.003639** | 9.86110 | **0.001890** |
| Tail, % | 1.84642 | 0.175421 | 0.00281 | 0.957763 | 5.82805 | **0.016489** |
| Head&Midpiece_% | 39.0015 | **<0.00001** | 6.1941 | **0.013466** | 0.0060 | 0.938135 |
| Head&Tail_% | 1.18864 | 0.276649 | 21.43479 | **<0.00001** | 1.68354 | 0.195646 |
| Midpiece&Tail_% | 6.232933 | **0.013182** | 0.971133 | 0.325347 | 3.092941 | 0.079851 |
| Head&Midpiece&Tail_% | 1.30309 | 0.254737 | 4.65874 | **0.031844** | 0.00095 | 0.975421 |

Note

Significant (p<0.05) effects of factors are highlighted by bold text.

Abbreviations: DFI – DNA fragmentation index; TZI – teratozoospermia index; ERC – excess residual cytoplasm.
