# Supplementary material for: Impaired semen quality, an increase of sperm morphological defects and DNA fragmentation associated with environmental pollution in urban population of young men from Western Siberia, Russia
Source: PLoS One. 2021 Oct 22;16(10):e0258900. doi: 10.1371/journal.pone.0258900 (PMC8535459; doi:10.1371/journal.pone.0258900)
Supplement: S4 Table — Significant (p<0.05) effects of factors are highlighted by bold text. Abbreviations: DFI–DNA fragmentation index; TZI–teratozoospermia index; ERC–excess residual cytoplasm. (DOCX) [file pone.0258900.s004.docx]

**S4 Table.**

The effects of smoking on sperm quality and sperm morphology

(ANCOVA results).

|  |  | Factors | | | | |
| --- | --- | --- | --- | --- | --- | --- |
|  | City |  | Smoking status | | Smoking status&City | |
| Parameters |  |  |  |  |  |  |
|  | F criterion | p value | F criterion | p value | F criterion | p value |
|  |  |  |  |  |  |  |
| Sperm count, mln | 12.99219 | **0.000345** | 6.14455 | **0.013521** | 1.14071 | 0.286034 |
| Sperm concentration, mln/ml | 8.66409 | **0.003401** | 1.81356 | 0.178712 | 0.68077 | 0.409728 |
| Progressive motility, % | 10.91140 | **0.001027** | 6.09892 | **0.013870** | 2.91707 | 0.088288 |
| Normal sperm, % | 27.1258 | **<0.00001** | 1.1498 | 0.284122 | 1.1498 | 0.284122 |
| TZI | 56.663 | **<0.00001** | 7.659 | **0.005866** | 5.656 | **0.017784** |
| DFI, % | 1.93890 | 0.165006 | 6.04531 | **0.014611** | 1.45741 | 0.228466 |
| Amorphous head, % | 81.3463 | **<0.00001** | 1.0564 | 0.304544 | 0.3425 | 0.558644 |
| Pyriform head, % | 29.89063 | **<0.00001** | 0.56355 | 0.453199 | 0.00738 | 0.931577 |
| Elongated head, % | 36.80446 | **<0.00001** | 3.51984 | 0.061240 | 0.33886 | 0.560758 |
| Round head, % | 50.29488 | **<0.00001** | 0.05828 | 0.809342 | 0.51280 | 0.474276 |
| Large head, % | 0.039229 | 0.843080 | 0.196285 | 0.657934 | 2.323864 | 0.128056 |
| Small head, % | 4.78946 | **0.029112** | 0.70498 | 0.401530 | 0.65908 | 0.417283 |
| Double head, % | 6.789953 | **0.009450** | 3.994963 | **0.046195** | 8.701351 | **0.003334** |
| Vacuolated head, % | 22.9663 | **<0.00001** | 3.4518 | 0.063789 | 1.2188 | 0.270149 |
| Abnormal acrosome, % | 13.9015 | **0.000215** | 1.7562 | 0.185729 | 1.4034 | 0.236733 |
| Bent_head, % | 0.9974 | 0.318447 | 0.6932 | 0.405498 | 2.2688 | 0.132657 |
| ERC, % | 11.8949 | **0.000612** | 3.3703 | 0.066994 | 1.0472 | 0.306655 |
| Asymmetrical neck insertion, % | 354.1220 | **<0.00001** | 6.7433 | **0.009696** | 0.6895 | 0.406756 |
| Thick mipiece, % | 12.7019 | **0.000402** | 0.0369 | 0.847816 | 2.5214 | 0.112962 |
| Thin midpiece, % | 8.74255 | **0.003261** | 0.88346 | 0.347724 | 1.83695 | 0.175941 |
| Double tail, % | 0.00950 | 0.922387 | 0.02829 | 0.866507 | 0.00005 | 0.994345 |
| Coiled tail,% | 2.3822 | 0.123376 | 0.9578 | 0.328238 | 4.4987 | **0.034429** |
| Short tail, % | 0.20948 | 0.647377 | 1.71502 | 0.190957 | 0.51444 | 0.473568 |
| **Abnormalities in different parts of spermatozoon** | | | | | | |
| Head, % | 156.350 | **<0.00001** | 8.161 | **0.004463** | 4.242 | **0.039967** |
| Midpiece,% | 13.65953 | **0.000244** | 0.65314 | 0.419391 | 0.17475 | 0.676108 |
| Tail, % | 178.6419 | **<0.00001** | 7.2334 | **0.007403** | 1.0362 | 0.309212 |
| Head&Midpiece_% | 1.2563 | 0.262908 | 0.2833 | 0.594770 | 3.9777 | **0.046667** |
| Head&Tail_% | 178.6419 | **<0.00001** | 7.2334 | **0.007403** | 1.0362 | 0.309212 |
| Midpiece&Tail_% | 1.2563 | 0.262908 | 0.2833 | 0.594770 | 3.9777 | **0.046667** |
| Head&Midpiece&Tail_% | 5.5657 | **0.018712** | 9.8393 | **0.001812** | 1.1292 | 0.288481 |

Note

Significant (p<0.05) effects of factors are highlighted by bold text.

Abbreviations: DFI – DNA fragmentation index; TZI – teratozoospermia index; ERC – excess residual cytoplasm.
