# Supplementary material for: Impaired semen quality, an increase of sperm morphological defects and DNA fragmentation associated with environmental pollution in urban population of young men from Western Siberia, Russia
Source: PLoS One. 2021 Oct 22;16(10):e0258900. doi: 10.1371/journal.pone.0258900 (PMC8535459; doi:10.1371/journal.pone.0258900)
Supplement: S5 Table — Significant (p<0.05) effects of factors are highlighted by bold text. Abbreviations: DFI–DNA fragmentation index; TZI–teratozoospermia index; ERC–excess residual cytoplasm. (DOCX) [file pone.0258900.s005.docx]

**S5 Table.**

**The effects of alcohol consumption on sperm quality and sperm morphology (ANCOVA results).**

|  |  | Factors | | | | |
| --- | --- | --- | --- | --- | --- | --- |
|  | City |  | Alcohole status | | Alcohole status&City | |
|  |  |  |  |  |  |  |
| Parameters | F criterion | p value | F criterion | p value | F criterion | p value |
| Sperm count, mln | 16.32545 | **0.000062** | 1.05105 | 0.305778 | 3.21838 | 0.073440 |
| Sperm concentration, mln/ml | 12.48579 | **0.000449** | 1.51036 | 0.219681 | 3.88044 | 0.049421 |
| Progressive motility, % | 12.15457 | **0.000534** | 2.34375 | 0.126440 | 3.03152 | 0.082297 |
| Normal sperm, % | 33.1825 | **<0.00001** | 2.5472 | 0.111143 | 6.1361 | 0.013586 |
| TZI | 46.713 | **<0.00001** | 3.352 | 0.067738 | 1.280 | 0.258367 |
| DFI, % | 4.04244 | **0.045427** | 0.14214 | 0.706480 | 0.05062 | 0.822174 |
| Amorphous head, % | 76.2065 | **<0.00001** | 0.5170 | 0.472474 | 0.0025 | 0.959869 |
| Pyriform head, % | 29.88682 | **<0.00001** | 0.00790 | 0.929229 | 0.46084 | 0.497555 |
| Elongated head, % | 36.06361 | **<0.00001** | 1.07379 | 0.300609 | 0.00008 | 0.992727 |
| Round head, % | 45.29818 | **<0.00001** | 0.34580 | 0.556774 | 0.52888 | 0.467430 |
| Large head, % | 0.684929 | 0.408303 | 0.141059 | 0.707395 | 0.067621 | 0.794943 |
| Small head, % | 2.71845 | 0.099843 | 0.94453 | 0.331600 | 0.06397 | 0.800436 |
| Double head, % | 0.090272 | 0.763960 | 0.533777 | 0.465377 | 2.937243 | 0.087199 |
| Vacuolated head, % | 22.4710 | **0.000003** | 3.6363 | 0.057125 | 1.0871 | 0.297626 |
| Abnormal acrosome, % | 8.4240 | **0.003872** | 0.9167 | 0.338809 | 0.2495 | 0.617666 |
| Bent_head, % | 3.2803 | 0.070737 | 1.8027 | 0.180014 | 7.3252 | 0.007040 |
| ERC, % | 11.6656 | **0.000690** | 0.3199 | 0.571945 | 0.8084 | 0.369055 |
| Asymmetrical neck insertion, % | 313.3490 | **<0.00001** | 1.1987 | 0.274135 | 0.2134 | 0.644298 |
| Thick mipiece, % | 9.7008 | **0.001951** | 0.5346 | 0.465027 | 0.5338 | 0.465373 |
| Thin midpiece, % | 5.27708 | **0.022034** | 0.13208 | 0.716441 | 0.00576 | 0.939542 |
| Double tail, % | 1.24586 | 0.264899 | 2.61201 | 0.106709 | 4.38473 | 0.036782 |
| Coiled tail,% | 0.3430 | 0.558382 | 0.6604 | 0.416806 | 0.0631 | 0.801801 |
| Short tail, % | 0.04733 | 0.827864 | 4.62151 | 0.032068 | 0.70612 | 0.401150 |
| Abnormalities in different parts of spermatozoon | | | | | | |
| Head, % | 126.8797 | **<0.00001** | 0.4891 | 0.484661 | 0.3250 | 0.568878 |
| Midpiece,% | 27.41167 | **<0.00001** | 1.66453 | 0.197609 | 3.51311 | 0.061487 |
| Tail, % | 21.13774 | **<0.00001** | 0.16611 | 0.683774 | 3.33833 | 0.068299 |
| Head&Midpiece_% | 166.4192 | **<0.00001** | 0.0046 | 0.945902 | 1.7997 | 0.180378 |
| Head&Tail_% | 0.3492 | 0.554856 | 2.2485 | 0.134394 | 0.2748 | 0.600357 |
| Midpiece&Tail_% | 4.420207 | **0.036032** | 0.250086 | 0.617242 | 1.794891 | 0.180960 |
| Head&Midpiece&Tail_% | 8.4594 | **0.003799** | 1.0691 | 0.301660 | 3.1090 | 0.078492 |

Note

Significant (p<0.05) effects of factors are highlighted by bold text.

Abbreviations: DFI – DNA fragmentation index; TZI – teratozoospermia index; ERC – excess residual cytoplasm.
