# Supplementary material for: Impaired semen quality, an increase of sperm morphological defects and DNA fragmentation associated with environmental pollution in urban population of young men from Western Siberia, Russia
Source: PLoS One. 2021 Oct 22;16(10):e0258900. doi: 10.1371/journal.pone.0258900 (PMC8535459; doi:10.1371/journal.pone.0258900)
Supplement: S6 Table — Results based on raw data. Analysis of variance used to compare all parameters. Significant (p<0.05) differences between groups are highlighted by bold text. Abbreviations: SD—standard deviation; (5–95) - 5th–95th percentile; DFI–DNA fragmentation index; TZI–teratozoospermia index; ERC–excess residual cytoplasm. (DOCX) [file pone.0258900.s006.docx]

**S6 Table**

## The effects of alcohol consumption on sperm quality and sperm morphology

|  | Aclohole consumption |  |  |  |  |
| --- | --- | --- | --- | --- | --- |
| Parameters | No(n=132) |  | Yes(n=403) |  | P value |
|  |  |  |  |  |  |
|  | Mean (SD) | Median(5-95) | Mean (SD) | Median(5-95) |  |
| Sperm count, mln | 207.11  (158.62) | 179.21  (12.35-450.5) | 214.52  (207.79) | 171.61  (19.75-467.17) | 0.870071 |
| Sperm concentration, mln/ml | 55.01  (41.75) | 43.06  (4.37-126.99) | 57.43  (41.64) | 48.89  (6.81-142.55) | 0.611700 |
| Progressive motility, % | 43.36  (26.18) | 39.53  (2.57-87.52) | 45.77  (26.75) | 44.7  (4.13-89.23) | 0.260740 |
| Normal sperm, % | 6.97(3.32) | 7.19  (1.50-13.75) | 7.29(3.09) | 7.25(2.5-12.25) | 0.229183 |
| TZI | 1.5(0.13) | 1.47(1.33-1.75) | 1.48(0.13) | 1.47(1.31-1.73) | 0.092053 |
| DFI, % | 9.48(7.02) | 7.01(2.4-24.39) | 9.67(7.83) | 7.26(2.72-29.10) | 0.909699 |
| Amorphous head, % | 63.78(15.33) | 66(33.50-85.0) | 62.77(14.51) | 64.50(35.5-83.0) | 0.468591 |
| Pyriform head, % | 9.99(10.38) | 6.75(0.50-34.5) | 9.62(10.12) | 6.50(0.5-33.5) | 0.940413 |
| Elongated head, % | 10.72(9.86) | 9.0(1.0-31.50) | 11.43(9.18) | 8.50(1.5-28.5) | 0.282619 |
| Round head, % | 1.8(2.31) | 1.0(0.00-6.50) | 1.63(2.08) | 1.00(0.0-5.5) | 0.420432 |
| Large head, % | 0.14(0.28) | 0.00(0.00-0.50) | 0.13(0.31) | 0.00(0.0-0.5) | 0.858755 |
| Small head, % | 0.51(0.79) | 0.0(0.0-2.0) | 0.57(0.85) | 0.50(0.0-2.5) | 0.359228 |
| Double head, % | 0.03(0.16) | 0.0(0.0-0.0) | 0.06(0.21) | 0.0(0.0-0.5) | 0.307318 |
| Vacuolated head, % | **10.13(6.80)** | 8.75(1.50-22.5) | **11.11(7.04)** | 9.50(2.5-23.5) | **0.019454** |
| Abnormal acrosome, % | 16.82(10.69) | 14.50(5.50-40) | 18.22(11.42) | 15.00(5.5-42.0) | 0.272352 |
| Bent_head, % | 5.89(3.97) | 5.0(1.0-13.3) | 5.84(4.18) | 4.50(1.5-14.5) | 0.458313 |
| ERC, % | 7.91(4.62) | 7.00(2.0-16.0) | 7.71(4.68) | 6.50(2-15.5) | 0.627374 |
| Asymmetrical neck insertion, % | 17.82(7.66) | 17.75(6.0-31.0) | 18.46(8.29) | 18.00(6.5-33.0) | 0.369362 |
| Thick mipiece, % | 6.97(4.00) | 6.5(1.5-14.5) | 6.73(3.63) | 6.00(2.0-13.25) | 0.586720 |
| Thin midpiece, % | 0.99(1.12) | 0.75(0.0-3.0) | 1.05(1.25) | 0.50(0.0-3.0) | 0.754813 |
| Double tail, % | 1.44(1.33) | 1.0(0.0-4.0) | 1.26(1.16) | 1.00(0.0-3.5) | 0.144501 |
| Coiled tail,% | 10.61(5.73) | 9.50(3.5-21.5) | 10.21(5.96) | 9.00(3.5-21.75) | 0.289071 |
| Short tail, % | 2.82(2.42) | 2.25(0.5-7) | 2.46(2.18) | 2.00(0.0-6.5) | 0.063197 |
| Abnormalities in different parts of spermatozoon | | | | | |
| Head, % | 45.9(10.10) | 45.25(29-62) | 46.18(9.97) | 46.50(29-62.5) | 0.412459 |
| Midpiece,% | 3.81(2.95) | 3.00(0.5-9.5) | 4.15(2.85) | 3.50(0.5-9.5) | 0.290680 |
| Tail, % | 1.37(1.59) | 1.00(0.0-5.0) | 1.32(1.34) | 1.00(0.0-3.5) | 0.979122 |
| Head&Midpiece, % | 28.39(8.92) | 27.00(16-43) | 28.66(8.47) | 28.50(16.0-43.5) | 0.861467 |
| Head&Tail, % | 9.67(4.78) | 9.00(3.5-19.5) | 9.01(5.16) | 8.00(3-18) | 0.103707 |
| Midpiece&Tail, % | 0.28(0.40) | 0.0(0.0-1.0) | 0.29(0.48) | 0.00(0-1.5) | 0.347824 |
| Head&Midpiece&Tail, % | 3.51(2.87) | 3.0(0.5-9.75) | 3.27(2.41) | 2.50(0.5-8.0) | 0.491338 |

*Note.* Results based on raw data. Analysis of variance used to compare all parameters. Significant (p<0.05) differences between groups are highlighted by bold text.

Abbreviations: SD - standard deviation; (5–95) - 5th–95th percentile; DFI – DNA fragmentation index; TZI – teratozoospermia index; ERC – excess residual cytoplasm.
