# Supplementary material for: Impaired semen quality, an increase of sperm morphological defects and DNA fragmentation associated with environmental pollution in urban population of young men from Western Siberia, Russia
Source: PLoS One. 2021 Oct 22;16(10):e0258900. doi: 10.1371/journal.pone.0258900 (PMC8535459; doi:10.1371/journal.pone.0258900)
Supplement: S7 Table — Significant (p<0.05) effects of factors are highlighted by bold text. Abbreviations: DFI–DNA fragmentation index; TZI–teratozoospermia index; ERC–excess residual cytoplasm. (DOCX) [file pone.0258900.s007.docx]

**S7 Table.**

The effects of obesity on sperm quality and sperm morphology (ANCOVA results).

|  |  | Factors | | | | |
| --- | --- | --- | --- | --- | --- | --- |
|  | City |  | Obesity status | | Obesity status&City | |
| Parameters |  |  |  |  |  |  |
|  | F criterion | p value | F criterion | p value | F criterion | p value |
|  |  |  |  |  |  |  |
| Sperm count, mln | 6.16690 | **0.013357** | 4.49475 | **0.011642** | 0.61479 | 0.541178 |
| Sperm concentration, mln/ml | 4.30433 | **0.038548** | 4.57507 | **0.010759** | 0.38666 | 0.679536 |
| Progressive motility, % | 6.07774 | **0.014038** | 3.59876 | **0.028098** | 0.82851 | 0.437325 |
| Normal sperm, % | 0.3067 | 0.736011 | 0.3067 | 0.736011 | 2.3910 | 0.092625 |
| TZI | 14.351 | **0.000171** | 0.012 | 0.988488 | 0.460 | 0.631342 |
| DFI, % | 4.80672 | **0.029271** | 0.56671 | 0.568115 | 0.74247 | 0.476981 |
| Amorphous head, % | 43.3115 | **<0.00001** | 1.3582 | 0.258098 | 1.1024 | 0.332899 |
| Pyriform head, % | 11.56529 | **0.000728** | 1.31640 | 0.269064 | 0.23155 | 0.793391 |
| Elongated head, % | 27.43676 | **<0.00001** | 2.23346 | 0.108269 | 1.00469 | 0.366926 |
| Round head, % | 23.81427 | **<0.00001** | 0.47469 | 0.622369 | 0.14577 | 0.864393 |
| Large head, % | 0.647489 | 0.421410 | 0.387892 | 0.678698 | 0.210713 | 0.810081 |
| Small head, % | 2.28423 | 0.131353 | 0.05852 | 0.943164 | 0.03932 | 0.961443 |
| Double head, % | 0.08878 | 0.765869 | 1.14631 | 0.318676 | 0.29884 | 0.741816 |
| Vacuolated head, % | 7.1865 | **0.007598** | 0.9477 | 0.388342 | 0.5745 | 0.563387 |
| Abnormal acrosome, % | 15.3407 | **0.000103** | 3.4798 | 0.031593 | 3.6667 | 0.026280 |
| Bent_head, % | 0.19728 | 0.657126 | 0.70463 | 0.494801 | 0.83804 | 0.433189 |
| ERC, % | 6.4555 | **0.011375** | 0.2730 | 0.761213 | 0.2088 | 0.811659 |
| Asymmetrical neck insertion, % | 169.3862 | **<0.00001** | 0.3480 | 0.706248 | 0.1374 | 0.871650 |
| Thick mipiece, % | 4.7016 | **0.030626** | 0.2047 | 0.814931 | 0.4953 | 0.609693 |
| Thin midpiece, % | 1.06015 | 0.303700 | 0.21979 | 0.802767 | 0.34199 | 0.710530 |
| Double tail, % | 1.74040 | 0.187717 | 1.72401 | 0.179452 | 1.57553 | 0.207967 |
| Coiled tail,% | 0.2040 | 0.651729 | 0.3333 | 0.716699 | 0.8967 | 0.408597 |
| Short tail, % | 0.00730 | 0.931960 | 1.31687 | 0.268940 | 1.06319 | 0.346163 |
| Abnormalities in different parts of spermatozoon | | | | | | |
| Head, % | 58.9509 | **<0.00001** | 0.4947 | 0.610074 | 0.2390 | 0.787478 |
| Midpiece,% | 21.03192 | **<0.00001** | 0.89064 | 0.411069 | 0.75957 | 0.468427 |
| Tail, % | 15.94579 | **0.000075** | 1.40315 | 0.246827 | 2.28031 | 0.103359 |
| Head&Midpiece_% | 80.7968 | **<0.00001** | 0.3346 | 0.715825 | 0.3039 | 0.738112 |
| Head&Tail_% | 0.2779 | 0.598321 | 1.6196 | 0.199064 | 0.5466 | 0.579279 |
| Midpiece&Tail_% | 2.473313 | 0.116453 | 0.296456 | 0.743585 | 0.163170 | 0.849494 |
| Head&Midpiece&Tail_% | 3.97634 | **0.046709** | 0.61690 | 0.540042 | 0.43909 | 0.644879 |

Note

Significant (p<0.05) effects of factors are highlighted by bold text.

Abbreviations: DFI – DNA fragmentation index; TZI – teratozoospermia index; ERC – excess residual cytoplasm.
