# Supplementary material for: Impaired semen quality, an increase of sperm morphological defects and DNA fragmentation associated with environmental pollution in urban population of young men from Western Siberia, Russia
Source: PLoS One. 2021 Oct 22;16(10):e0258900. doi: 10.1371/journal.pone.0258900 (PMC8535459; doi:10.1371/journal.pone.0258900)
Supplement: S8 Table — Results based on raw data. Analysis of variance used to compare all parameters. Significant (p<0.05) differences between groups are highlighted by bold text. Abbreviations: SD—standard deviation; (5–95) - 5th–95th percentile; DFI–DNA fragmentation index; TZI–teratozoospermia index; ERC–excess residual cytoplasm. (DOCX) [file pone.0258900.s008.docx]

**S8 Table.** **The effects of obesity on sperm quality and sperm morphology.**

|  | Normal weght(n=377) |  | Overweght (n=126) | |  | | Obesity (n=30) |  |  |
| --- | --- | --- | --- | --- | --- | --- | --- | --- | --- |
| Parameters |  |  |  | |  | |  |  |  |
|  | Mean(SD) | Median(5-95) | Mean(SD) | | Median(5-95) | | Mean(SD) | Median(5-95) | P value |
|  |  |  |  | |  | |  |  |  |
| Normal sperm, % | 7.16(3.05) | 7.25(2.35-12.25) | 7.19(3.22) | | 7.25(1.75-11.75) | | 7.64(3.86) | 7.99(1.06-14.5) | 0.736011 |
| TZI | 1.49(0.12) | 1.47(1.32-1.73) | 1.48(0.13) | | 1.46(1.32-1.76) | | 1.49(0.14) | 1.48(1.31-1.79) | 0.988488 |
| DFI, % | 9.86(7.6) | 7.60(2.82-26.25) | 9.28(7.49) | | 6.80(2.38-27.35) | | 8.9(9.35) | 4.96(1.81-32.76) | 0.568115 |
| Amorphous head, % | 63.45(14.62) | 65.00(34.0-84.0) | 60.96(14.47) | | 63.00(34.0-83.0) | | 65.59(16.7) | 68.50(28.85-84.16) | 0.258098 |
| Pyriform head, % | 9.76(10.3) | 6.50(0.5-34.5) | 10.01(10.07) | | 6.50(1.0-35.3) | | 8.41(9.28) | 4.00(0.99-23.50) | 0.269064 |
| Elongated head, % | 10.88(8.94) | 8.5(1.0-28.5) | 12.80(10.05) | | 10.00(1.5-32.5) | | 10(10.82) | 6.25(1.00-29.00) | 0.108269 |
| Round head, % | 1.75(2.23) | 1.00(0-6.5) | 1.52(1.97) | | 1.0(0.00-5.00) | | 1.26(1.68) | 0.50(0.0-5.0) | 0.622369 |
| Large head,% | 0.13(0.29) | 0.00(0-0.5) | 0.13(0.33) | | 0.00(0.0-0.5) | | 0.18(0.31) | 0.00(0.0-1.0) | 0.678698 |
| Small head, % | 0.56(0.81) | 0.50(0-2) | 0.55(0.86) | | 0.25(0.0-2.5) | | 0.52(0.96) | 0.0(0.0-2.97) | 0.943164 |
| Double head, % | 0.04(0.2) | 0.00(0.0-0.5) | 0.06(0.19) | | 0.00(0.0-0.5) | | 0.1(0.27) | 0.0(0.0-0.9) | 0.318676 |
| Vacuolated head, % | 10.80(7.11) | 9.25(2.25-23) | 10.84(6.54) | | 9.75(2-22) | | 12.32(7.31) | 10.51(2.5-30.0) | 0.388342 |
| Abnormal acrosome, % | 17.24(10.77) | 14.50(5.5-39.0) | 19.63(12.32) | | 15.75(5.5-42.67) | | 18.83(12.2) | 16.25(6.0-40.35) | 0.061593 |
| Bent_head, % | 5.74(3.86) | 5.0(1.0-13.5) | 6.20(4.85) | | 5.00(1.5-13.5) | | 5.94(4.17) | 4.50(2.0-16.83) | 0.494801 |
| ERC, % | 7.73(4.43) | 7.0(2-16) | 7.57(4.48) | | 6.50(2.0-15.0) | | 9.34(7.27) | 7.75(2.0-29.8) | 0.761213 |
| Asymmetrical neck insertion, % | 18.52(8.06) | 18.5(6.75-33.0) | 18.12(8.31) | | 17.50(6.5-32) | | 17.08(8.17) | 15.75(5.5-29.5) | 0.706248 |
| Thick mipiece, % | 6.77(3.74) | 6.0(2.0-13.5) | 6.89(3.69) | | 6.50(2-13.5) | | 6.65(3.83) | 6.25(2.5-12) | 0.814931 |
| Thin midpiece, % | 1.07(1.29) | 0.75(0.0-3.5) | 1.00(1.09) | | 0.50(0-2.67) | | 0.85(0.9) | 0.50(0-2.97) | 0.802767 |
| Double tail, % | 1.25(1.17) | 1.00(0-3.5) | 1.35(1.14) | | 1.00(0.00-3.50) | | 1.78(1.71) | 1.50(0-5.5) | 0.179452 |
| Coiled tail,% | 10.16(5.60) | 9.00(3.5-21.0) | 10.78(6.82) | | 8.75(3.5-23.5) | | 10.5(5.59) | 10.45(4.5-17.5) | 0.716699 |
| Short tail, % | 2.46(2.10) | 2.00(0.0-6.0) | 2.78(2.48) | | 2.00(0.00-8.00) | | 2.74(2.92) | 2.25(0.5-11.54) | 0.268940 |
| Abnormalities in different parts of spermatozoon | | | | | | | | | |
| Head, % | 46.26(9.87) | 46.00(29.5-63.0) | 45.7(10.13) | 46.25(26.5-62) | | 45.19(10.95) | | 46.50(23-60) | 0.610074 |
| Midpiece,% | 3.96(2.79) | 3.50(0.5-9.5) | 4.26(3.14) | 3.50(0.5-9.5) | | 4.58(2.82) | | 3.73(0-8.5) | 0.411069 |
| Tail, % | 1.34(1.43) | 1.00(0.0-4.0) | 1.36(1.38) | 1.00(0-3.5) | | 1.07(1.27) | | 0.75(0-3.5) | 0.246827 |
| Head&Midpiece_% | 28.85(8.45) | 28.50(15.5-43) | 28.16(8.19) | 28.00(16.5-43) | | 28.07(11.01) | | 26.25(15-50) | 0.715825 |
| Head&Tail_% | 8.93(4.67) | 8.00(3-18) | 9.67(6.1) | 8.25(3.0-20.0) | | 10.32(5.18) | | 9.5(4.5-17.5) | 0.199064 |
| Midpiece&Tail_% | 0.27(0.45) | 0.00(0-1) | 0.33(0.49) | 0.00(0.0-1.5) | | 0.28(0.47) | | 0.00(0.0-1.0) | 0.743585 |
| Head&Midpiece&Tail_% | 3.29(2.41) | 2.50(0.5-8) | 3.49(2.94) | 2.50(0.5-9.0) | | 3.32(2.23) | | 2.75(1.0-8.65) | 0.540042 |

*Note.* Results based on raw data. Analysis of variance used to compare all parameters. Significant (p<0.05) differences between groups are highlighted by bold text.

Abbreviations: SD - standard deviation; (5–95) - 5th–95th percentile; DFI – DNA fragmentation index; TZI – teratozoospermia index; ERC – excess residual cytoplasm.
