# Supplementary material for: Impaired semen quality, an increase of sperm morphological defects and DNA fragmentation associated with environmental pollution in urban population of young men from Western Siberia, Russia
Source: PLoS One. 2021 Oct 22;16(10):e0258900. doi: 10.1371/journal.pone.0258900 (PMC8535459; doi:10.1371/journal.pone.0258900)
Supplement: S9 Table — Bold text indicates statistically significant (p<0.05) correlation coefficients. ERC–excess residual cytoplasm. (DOCX) [file pone.0258900.s009.docx]

**S9 Table**.

**Correlations DFI with other semen parameters and sperm morphology.**

| Parameters | Spearman's correlation coefficients for DFI | | |
| --- | --- | --- | --- |
|  | Entire population | Population from | |
|  |  | Novosibirsk | Kemerovo |
| Sperm count, mln | **-0,27** | **-0,30** | -0,13 |
| Sperm concentration, mln/ml | **-0,30** | **-0,34** | **-0,22** |
| Progressive motility, % | **-0,46** | **-0,52** | **-0,34** |
| TZI | **0,38** | **0,33** | **0,33** |
| Normal sperm, % | **-0,36** | **-0,33** | **-0,32** |
| **Head defects** | | | |
| Amorphous, % | -0,09 | -0.07 | -0.08 |
| Pyriform, % | 0,07 | 0,05 | -0,04 |
| Elongated, % | **0,23** | **0,17** | 0,09 |
| Round, % | **0,19** | 0,10 | 0,15 |
| Large, % | -0,05 | -0,05 | 0,02 |
| Small, % | 0,10 | 0,12 | 0,15 |
| Double head % | **0,16** | **0,22** | 0,11 |
| Vacuolated, % | **0,26** | **0,17** | **0,24** |
| Abnormal acrosome, % | **0,35** | **0,43** | 0,19 |
| **Midpiece defects** | | | |
| Bent head, % | **0,18** | **0,21** | **0,28** |
| Assymetrical neck insertion % | **0,27** | **0,23** | -0,08 |
| Thick, % | 0,08 | 0,03 | 0,18 |
| Thin, % | **0,14** | **0,16** | 0,08 |
| **Tail defects** | | | |
| Double tail, % | 0,06 | 0,05 | 0,08 |
| Coiled tail,% | **0,21** | **0,17** | **0,34** |
| Short tail, % | **0,19** | **0,20** | 0,16 |
| **ERC** | | | |
| ERC, % | **0,16** | 0,13 | **0,20** |
| **Defects in different parts of spermatozoon** | | |  |
| Head, % | **-0,34** | **-0,25** | **-0,34** |
| Neck % | **-0,15** | **-0,25** | -0,11 |
| Tail % | -0,10 | -0,13 | 0,17 |
| Head&Neck % | **0,34** | **0,27** | **0,20** |
| Head&Tail % | **0,28** | **0,26** | **0,37** |
| Neck&Tail % | -0,06 | **-0,11** | 0,02 |
| Head&Neck&Tail % | **0,23** | **0,25** | 0,15 |

Note.

Bold text indicates statistically significant (p<0.05) correlation coefficients.

ERC – excess residual cytoplasm
