# Supplementary material for: Impaired semen quality, an increase of sperm morphological defects and DNA fragmentation associated with environmental pollution in urban population of young men from Western Siberia, Russia
Source: PLoS One. 2021 Oct 22;16(10):e0258900. doi: 10.1371/journal.pone.0258900 (PMC8535459; doi:10.1371/journal.pone.0258900)
Supplement: S10 Table — Bold text indicates significant (p<0.05) correlation coefficients. ERC–excess residual cytoplasm. (DOCX) [file pone.0258900.s010.docx]

**S10Table.**

Spearman's correlation between percentages of sperm morphology defects and other semen parameters.

|  | Sperm count | Sperm concentration | Progressive motility |
| --- | --- | --- | --- |
| TZI | **-0,39** | **-0,44** | **-0,56** |
| Normal sperm, % | **0,51** | **0,62** | **0,67** |
| Head defects | | | |
| Amorphous, % | 0,04 | -0,01 | 0,02 |
| Pyriform, % | **-0,13** | **-0,09** | **-0,14** |
| Elongated, % | **-0,17** | **-0,11** | **-0,18** |
| Round, % | **-0,10** | **-0,12** | **-0,15** |
| Large, % | 0,04 | 0,02 | -0,01 |
| Small, % | -0,01 | -0,03 | **-0,09** |
| Double head, % | -0,06 | **-0,09** | **-0,13** |
| Vacuolated, % | **-0,18** | **-0,12** | **-0,20** |
| Abnormal acrosome, % | **-0,29** | **-0,31** | **-0,38** |
| Bent head, % | **-0,31** | **-0,34** | **-0,37** |
| Assymetrical neck insertion, % | **-0,18** | **-0,15** | **-0,14** |
| Thick, % | **-0,18** | **-0,23** | **-0,25** |
| Thin, % | **-0,18** | **-0,18** | **-0,23** |
| **Tail defects** | | | |
| Double tail, % | **-0,16** | **-0,15** | **-0,15** |
| Coiled tail, % | **-0,13** | **-0,22** | **-0,36** |
| Short tail, % | **-0,34** | **-0,40** | **-0,45** |
| **ERC** | | | |
| ERC, % | **-0,15** | **-0,17** | **-0,23** |
| **Defects in different parts of spermatozoon** | | | |
| Head % | **0,26** | **0,29** | **0,38** |
| Neck % | **0,16** | **0,19** | **0,26** |
| Tail % | **0,18** | **0,14** | **0,14** |
| Head&Neck % | **-0,34** | **-0,35** | **-0,39** |
| Head&Tail % | **-0,18** | **-0,26** | **-0,42** |
| Neck&Tail % | 0,05 | -0,01 | -0,03 |
| Head&Neck&Tail % | **-0,41** | **-0,46** | **-0,54** |

Note.

Bold text indicates significant (p<0.05) correlation coefficients.

ERC – excess residual cytoplasm
