# Supplementary material for: Impaired semen quality, an increase of sperm morphological defects and DNA fragmentation associated with environmental pollution in urban population of young men from Western Siberia, Russia
Source: PLoS One. 2021 Oct 22;16(10):e0258900. doi: 10.1371/journal.pone.0258900 (PMC8535459; doi:10.1371/journal.pone.0258900)
Supplement: S11 Table — Bold text indicates significant (p<0.05) correlation coefficients. DH–double head; BH–bent head; ERC–excess residual cytoplasm; ANI–asymmetrical neck insertion; DT–double tail; CT–coiled tail; ST–short tail. (DOCX) [file pone.0258900.s011.docx]

**S11 Table.** **Spearman's correlation coefficients among percentages of different sperm morphology defects**

|  |  | **Head defects** | | | | | | | | | **Midpiece defects and ERC** | | | | | **Tail defects** | | |  |
| --- | --- | --- | --- | --- | --- | --- | --- | --- | --- | --- | --- | --- | --- | --- | --- | --- | --- | --- | --- |
|  |  | Amorphous | Pyriform | Elongated | Round | large | Small | DH | Vacuolated | Abnormal acrosome | BH | ERC | ANI | Thick | Thin | DT | ST |  | CT |
| **Head defects** | Amorphous | 1,00 | **-0,74** | **-0,84** | **0,20** | 0,00 | **0,14** | 0,05 | **-0,27** | -0,08 | **-0,13** | **-0,26** | **-0,29** | 0,04 | 0,01 | **0,11** | 0,08 |  | 0,01 |
|  | Pyriform | **-0,74** | 1,00 | **0,65** | **-0,31** | -0,01 | **-0,15** | -0,02 | **0,29** | -0,07 | **0,29** | **0,40** | 0,06 | -0,06 | 0,01 | -0,06 | 0,03 |  | 0,03 |
|  | Elongated | **-0,84** | **0,65** | 1,00 | **-0,24** | 0,00 | -0,09 | -0,02 | **0,30** | **0,28** | **0,15** | **0,24** | **0,25** | -0,06 | 0,04 | -0,07 | 0,03 |  | 0,01 |
|  | Round | **0,20** | **-0,31** | **-0,24** | 1,00 | -0,01 | **0,30** | 0,05 | -0,03 | **0,32** | -0,06 | -0,04 | **0,34** | **0,24** | 0,05 | 0,06 | **0,13** |  | 0,03 |
|  | large | 0,00 | -0,01 | 0,00 | -0,01 | 1,00 | 0,02 | 0,05 | 0,03 | -0,04 | 0,05 | 0,00 | 0,00 | -0,01 | 0,01 | 0,09 | 0,03 |  | 0,00 |
|  | Small | **0,14** | **-0,15** | -0,09 | **0,30** | 0,02 | 1,00 | **0,12** | -0,03 | **0,26** | 0,02 | 0,01 | -0,03 | **0,19** | **0,10** | 0,05 | **0,14** |  | 0,03 |
|  | DH | 0,05 | -0,02 | -0,02 | 0,05 | 0,05 | **0,12** | 1,00 | 0,08 | **0,14** | 0,09 | 0,09 | -0,01 | 0,04 | 0,06 | **0,12** | **0,11** |  | 0,01 |
|  | Vacuolated | **-0,27** | **0,29** | **0,30** | -0,03 | 0,03 | -0,03 | 0,08 | 1,00 | **0,13** | **0,16** | **0,26** | **0,23** | -0,09 | **0,15** | 0,04 | **0,16** |  | 0,07 |
|  | Abnormal  acrosome | -0,08 | -0,07 | **0,28** | **0,32** | -0,04 | **0,26** | **0,14** | **0,13** | 1,00 | **0,18** | **0,12** | **0,24** | **0,20** | **0,21** | 0,06 | **0,23** | **0,15** | |
| **Midpiece dsefects and ERC** | BH | **-0,13** | **0,29** | **0,15** | -0,06 | 0,05 | 0,02 | 0,09 | **0,16** | **0,18** | 1,00 | **0,37** | **-0,16** | **0,17** | **0,13** | 0,04 | **0,19** | **0,28** | |
|  | ERC | **-0,26** | **0,40** | **0,24** | -0,04 | 0,00 | 0,01 | 0,09 | **0,26** | **0,12** | **0,37** | 1,00 | **0,12** | **0,20** | **0,16** | **0,12** | **0,10** | **0,14** | |
|  | ANI | **-0,29** | 0,06 | **0,25** | **0,34** | 0,00 | -0,03 | -0,01 | **0,23** | **0,24** | **-0,16** | **0,12** | 1,00 | 0,06 | 0,08 | 0,04 | 0,02 |  | -0,05 |
|  | Thick | 0,04 | -0,06 | -0,06 | **0,24** | -0,01 | **0,19** | 0,04 | -0,09 | **0,20** | **0,17** | **0,20** | 0,06 | 1,00 | **0,17** | **0,15** | **0,15** | **0,13** | |
|  | Thin | 0,01 | 0,01 | 0,04 | 0,05 | 0,01 | **0,10** | 0,06 | **0,15** | **0,21** | **0,13** | **0,16** | 0,08 | **0,17** | 1,00 | 0,09 | **0,21** | **0,15** | |
| **Tail defects** | DT | **0,11** | -0,06 | -0,07 | 0,06 | 0,09 | 0,05 | **0,12** | 0,04 | 0,06 | 0,04 | **0,12** | 0,04 | **0,15** | 0,09 | 1,00 | 0,03 |  | -0,05 |
|  | CT | 0,01 | 0,03 | 0,01 | 0,03 | 0,00 | 0,03 | 0,01 | 0,07 | **0,15** | **0,28** | **0,14** | -0,05 | **0,13** | **0,15** | -0,05 | **0,32** | 1,00 | |
|  | ST | 0,08 | 0,03 | 0,03 | **0,13** | 0,03 | **0,14** | **0,11** | **0,16** | **0,23** | **0,19** | **0,10** | 0,02 | **0,15** | **0,21** | 0,03 | 1,00 | **0,32** | |

Note.

Bold text indicates significant (p<0.05) correlation coefficients.

DH – double head; BH – bent head; ERC – excess residual cytoplasm; ANI – asymmetrical neck insertion; DT – double tail; CT – coiled tail; ST – short tail.
